# Omentin-1 effects on mesenchymal stem cells: proliferation, apoptosis, and angiogenesis in vitro

**DOI:** 10.1186/s13287-017-0676-1

**Published:** 2017-10-10

**Authors:** Li Yin, Dan Huang, Xinxin Liu, Yongshun Wang, Jingjin Liu, Fang Liu, Bo Yu

**Affiliations:** 1 Key Laboratories of Education Ministry for Myocardial Ischemia Mechanism, The Second Affiliated Hospital of Harbin Medical University, 148 Baojian Road, Harbin, 150086 People’s Republic of China; 2 Department of Cardiology, The Second Affiliated Hospital of Harbin Medical University, 148 Baojian Road, Harbin, 150086 People’s Republic of China

**Keywords:** Omentin-1, Mesenchymal stem cells, Proliferation, Apoptosis, Reactive oxygen species, Mitochondrial apoptosis pathways, Angiogenesis

## Abstract

**Background:**

Mesenchymal stem cells (MSCs) are emerging as an extremely promising therapeutic agent for tissue repair. However, limitations exist such as the low numbers of MSCs obtained from donors, and the poor survival and function of donor cells. Omentin-1, a new fat depot-specific secretory adipokine, exerts proproliferation, prosurvival, and proangiogenic functions in certain cells via an Akt-dependent mechanism; however, little is known about the influence of omentin-1 on MSCs.

**Methods:**

MSCs were isolated from 60–80 g donor rats. Cell proliferation was assessed with CCK-8 and EdU assay. Cell cycle, apoptosis ratio, reactive oxygen species concentration, and mitochondrial membrane potential were detected by flow cytometry. Hoechst 33342 dye was used to assess morphological changes of apoptosis. Expression levels of Akt, FoxO3a, GSK-3β, and apoptosis- and cell cycle-associated proteins were detected by Western blotting. Tube formation assay was used to test the angiogenesis role of conditioned medium from MSCs in vitro. The cytokine secretion was assessed by ELISA.

**Results:**

After treatment with omentin-1 (100–800 ng/ml), MSCs displayed a higher proliferative capacity with an increasing number of cells in the S and G2 phase of the cell cycle. Moreover, omentin-1 preconditioning for 1 h could protect MSCs against H_2_O_2_-induced apoptosis in a concentration-dependent manner. Furthermore, omentin-1 pretreatment reduced the excessive reactive oxygen species. Western blots revealed that increased Bcl-2 and decreased Bax appeared in MSCs after omentin-1 incubation, which inhibited the mitochondrial apoptosis pathways with evidence showing inhibition of caspase-3 cleavage and preservation of mitochondrial membrane potential. Omentin-1 could enhance angiogenic growth factor secretion and elevate the ability of MSCs to stimulate tube formation by human umbilical vein endothelial cells (HUVECs). Furthermore, omentin-1 enhanced Akt phosphorylation; however, blockade of the PI3K/Akt pathway with an inhibitor, LY294002 (20 μM), suppressed the above beneficial effects of omentin-1.

**Conclusion:**

Omentin-1 can exert beneficial effects on MSCs by promoting proliferation, inhibiting apoptosis, increasing secretion of angiogenic cytokines, and enhancing the ability for stimulating tube formation by HUVECs via the PI3K/Akt signaling pathway. Thus, omentin-1 may be considered a candidate for optimizing MSC-based cell therapy.

## Background

Mesenchymal stem cells (MSCs) are adult stem cells and have attracted great interest for cell-based therapeutic strategies for tissue injury, such as myocardial infarction [1], stroke [2], and hind limb ischemia [3], because of their easy preparation, immunologic privilege, and ethical advantage. Unfortunately, the insufficient numbers of such MSCs obtained from donors [4], the low survival in the harsh environment, and the poor function of donor cells [5, 6] has limited their therapeutic potential. Remarkably, PI3K/Akt-related signaling pathways are known to function as core mechanisms by regulating multiple cellular behaviors of MSCs such as proliferation [7, 8], survival [9, 10], proangiogenesis [11], cytokine production [12], and so on. Therefore, optimized strategies to activate the PI3K/Akt pathway in MSCs need to be developed for better outcomes in stem cell therapy.

Omentin-1, referred to as intelectin-1, is a new fat depot-specific secretory adipokine that is a hydrophilic protein with a molecular weight of 34 kDa composed of 313 amino acids [13]; it has well-established effects on the regulation of insulin sensitivity [14], modulation of energy metabolism [15], and distribution of body fat [14, 16]. Omentin-1 is mainly expressed in visceral adipose tissue rather than in subcutaneous adipose tissue. Moreover, plasma omentin-1 levels in a healthy individual are reported to be roughly 370 ± 20 ng/mL [17], but are significantly reduced in obese patients, those with impaired glucose tolerance, and, particularly, in patients with type 2 diabetes mellitus [17–19] and cardiovascular disorders such as atherosclerosis [20] and ischemic heart disease [21]. Current data suggest that omentin-1 has protective effects on the cardiovascular system [22, 23]. Maruyama and colleagues [22] confirmed that, in a hind limb ischemia mouse model, omentin-1 enhanced blood flow recovery and capillary density in ischemia limbs of wild-type mice, and at the cellular level omentin-1 increased endothelial cell differentiation into vascular-like structures and lowed their apoptotic activity via PI3K/Akt-dependent signaling. Recently, Kataoka et al. [23] demonstrated that omentin-1 ameliorates acute ischemia injury in the heart by suppressing cardiomyocyte apoptosis through AMP-activated protein kinase (AMPK) and the Akt signaling pathway. However, to our knowledge, there has been no visual evidence of the protective effects of omentin-1 on the biological functions of MSCs. Therefore, we designed the present study to explore the potential role of omentin-1 on the proliferation, survival, angiogenesis, and cytokine production of MSCs and to elucidate whether the modulatory role of omentin-1 in MSCs was due to the activation of the PI3K/Akt signaling pathway.

## Methods

### Cell isolation and cultivation

MSCs were isolated from the bone marrow of Sprague-Dawley (SD) rats (weighing 60–80 g), as previously described [24, 25]. All of the SD rats were obtained from the Laboratory Animal Science Department, the Second Affiliated Hospital of Harbin Medical University (Harbin, China). All of the study procedures were approved by the Institutional Animal Care and Use Committee of Harbin Medical University (reference no. KY2016-180). This study was conducted in compliance with the Guide for the Care and Use of Laboratory Animals published by the National Academy Press (National Institutes of Health, revised in 1996). Briefly, the femurs and tibias were removed from the SD rats and the bone marrow was washed out using 10 ml of Dulbecco's modified Eagle’s medium/Nutrient Mixture F-12 (DMEM/F12; Gibco, Grand Island, NY, USA) with 1% penicillin/streptomycin (Beyotime Institute of Biotechnology, Nantong, China). The cells were centrifuged at 300 × g for 5 min. The resulting cell pellets were resuspended in 6 ml of DMEM/F12 supplemented with 10% fetal bovine serum (Gibco) and 1% penicillin/streptomycin and plated in a 25 cm^2^ plastic flask at 37 °C in a humidified atmosphere containing 5% CO_2_ to allow the adherence of the MSCs. After culturing for 3 days, the medium was changed, and the nonadherent cells were removed. The medium was replaced every 2 days. Upon reaching 80–90% confluence, the adherent cells were released from the dishes using 0.25% trypsin (Beyotime Institute of Biotechnology) and expanded at a dilution of 1:2 or 1:3. All subsequent experiments were performed using MSCs from passages 3–5.

Human umbilical vein endothelial cells (HUVECs) (American Type Culture Collection (ATCC), Manassas, VA, USA) were cultured in a 25 cm^2^ plastic flask in endothelial basal medium (EBM-2; Lonza) with 5% fetal bovine serum and 1% penicillin/streptomycin. The medium was replaced every 2 days. Upon reaching 80–90% confluence, the cells were released from the dishes using 0.25% trypsin (Beyotime Institute of Biotechnology), expanded at a dilution of 1:2 or 1:3 and used for experiments from passages 3–4.

### Cell proliferation assay

Cell proliferation was assessed with the cell counting kit-8 (CCK-8) assay (Beyotime Institute of Biotechnology) and 5-ethynyl-2’-deoxyuridine (EdU) proliferation assay (RiboBio Co., China). For the CCK-8 test, cells were plated onto 96-well plates (3 × 10^3^ cells/well) with omentin-1 (0–800 ng/ml; Omentin Human Recombinant; Prospect, Ness-Ziona, Israel) in a triplicate pattern. Assays were performed from 1 to 7 days after plating with the addition of 100 μl of fresh medium in 10 μl of CCK-8 solution for another 2 h at 37 °C. The optical density (OD) at 450 nm was measured. The assay was repeated three times.

For the EdU assay, cells were incubated with omentin-1 (800 ng/ml) for 5 days in 96-well plates, and then assessed using an EdU assay kit (RiboBio Co., China) according to the manufacturer's instructions. The EdU-positive cells were viewed under fluorescence microscopy (DMI4000B; Leica, Wetzlar, Germany) and the number calculated by counting at least three random separate fields.

### Cell cycle assay

The MSCs were cultured in serum-free medium overnight to synchronize the cell cycle before treatment. After treatment with omentin-1 (0–800 ng/ml) for 5 days, MSCs were dissociated into single cells with trypsin and fixed with prechilled 70% ethanol. These cells were stained with the Cycletest™ Plus kit (BD Biosciences, San Jose, CA, USA) according to the manufacturer’s instruction. Cell cycle analyses were carried out with flow cytometry (FACSCanto II), and data were analyzed using BD FACSDiva software (Becton-Dickinson, San Jose, CA, USA).

### Cell apoptosis assay

We used hydrogen peroxide and serum deprivation to induce MSC apoptosis. In brief, after cells were washed with phosphate-buffered saline (PBS), the culture medium was replaced with serum-free DMEM/F12 supplemented with 400 μM H_2_O_2_ and then the cells were placed at 37 °C for 6 h. For omentin-1 protection experiments, omentin-1 (0–800 ng/ml) was added to the MSC medium for 1 h before H_2_O_2_ treatment and then continuously incubated with 400 μM H_2_O_2_ for 6 h.

Cell death was assessed using the Annexin V-FITC/propidium iodide (PI) Apoptosis Detection kit (BD Biosciences) and Hoechst 33342 stain (Beyotime Institute of Biotechnology). According to the manufacturer's instructions for the Annexin V-FITC/PI Apoptosis Detection Kit, cells were harvested, washed in ice-cold PBS, and resuspended in 300 μl of binding buffer. Five microliters of Annexin V-FITC solution was added to the cells and incubated for 30 min at 4 °C in the dark. This was followed by further incubation with 5 μL PI for 5 min, and then they were analyzed immediately by bivariate flow cytometry using a BD FACSCanto cytometer equipped with FACSDiva Pro software (Becton-Dickinson, San Jose, CA, USA). Approximately 1–5 × 10^5^ cells were analyzed in each sample.

The chromatin dye Hoechst 33342 was used to assess the morphological changes of apoptosis. MSCs were fixed for 30 min in PBS containing 1% glutaraldehyde at room temperature, then washed twice with PBS and exposed to 5 mg/ml of Hoechst 33342 for 30 min at room temperature. The cells were then observed with a fluorescence microscope. Apoptotic cells were characterized by morphological alterations, such as condensed nuclei and cell shrinkage.

### Flow cytometry analysis of mitochondrial membrane potential (MMP or ΔΨm) and reactive oxygen species (ROS) levels

The loss of ΔΨm was determined using the JC-1 Mitochondrial Membrane Potential assay kit (Beyotime Institute of Biotechnology). Briefly, cells were washed with ice-cold PBS and then stained with 2.5 g/ml JC-1 for 30 min at 37 °C. After being washed with binding buffer, the cells were analyzed by flow cytometry (FACSCanto II). Results are presented as relative monomer-to-aggregate (green/red) fluorescence intensity ratio.

Cellular ROS levels were determined using a reactive oxygen species (ROS) assay kit (Beyotime Institute of Biotechnology). Briefly, the cells were incubated with the diluted fluoroprobe 2',7'-dichlorodihydrofluorescein diacetate (DCFH-DA; Beyotime Institute of Biotechnology) for 20 min at 37 °C with slight shaking every 5 min. After washing with serum-free culture medium, the cells were collected and examined by flow cytometry (FACSCanto II) at excitation and emission wavelengths of 488 and 525 nm, respectively.

### Western blot analysis

At the end of the treatment period, the MSCs were harvested and lysed with ice-cold RIPA lysis buffer, and the homogenate was centrifuged at 12,000 × g for 10 min at 4 °C. Total protein in the supernatant was quantified using a BCA Protein assay kit, and an aliquot (30–50 μg) from each sample was separated by 12% sodium dodecyl sulfate-polyacrylamide gel electrophoresis (SDS-PAGE). The protein band was transferred onto polyvinylidene difluoride (PVDF) membranes blocked with 8% fat-free milk in Tris-buffered saline (TBS) with 0.5% Tween-20 for 60 min at 37 °C, followed by treatment with the following primary antibodies at 4 °C overnight: rabbit monoclonal against Akt (cst-4691 s), phosphorylated Akt (p-Akt (Ser473); cst-4060 s, p-Akt (Thr308); cst-4056 s), Bax (cst-2772 s), Bcl-2 (cst-2876 s), and caspase-3 (cst-9662 s) (all from Cell Signaling Technology, Danvers, MA, USA), phosphorylated FoxO3a (phospho-FoxO3a (Thr32); AF605), FoxO3a (AF609), phosphorylated GSK-3β (phospho-GSK-3β (Ser9); AG753), GSK-3β (AG751), p21 (AP021), and p27 (AP027) (all from Beyotime Institute of Biotechnology), cyclin D1 (sc-70899) and cyclin E (sc-377100) (both from Santa Cruz Biotechnology, CA, USA), and mouse polyclonal antibody against β-actin (TA-09; Zhongshan Golden Bridge Biotechnology, Beijing, China). After washing in TBS with Tween-20 (TBS-T) buffer, the membranes were further incubated with horseradish peroxidase-conjugated anti-mouse (ZB-2305; Zhongshan Goldenbridge Biotechnology) and anti-rabbit (sc-2357) secondary antibodies (Santa Cruz Biotechnology) for 60 min at 37 °C. Subsequently, the membranes were washed in TBS-T solution three times, followed by the addition of TBS solution, and visualized using the ECL chemiluminescence detection system with BeyoECL Plus (Beyotime Institute of Biotechnology). Densitometric analysis of the protein bands was carried out using Quantity One software (Bio-Rad, Hercules, CA, USA).

### In vitro tube formation assay

The effect of MSC-conditioned medium on capillary-like tube formation on Matrigel (BD Bioscience) in vitro was evaluated. Conditioned media were collected from MSCs pretreated with omentin-1 or MSCs alone. To avoid the direct influence of omentin-1 on the capillary-like tube formation, after treatment with omentin-1 (800 ng/ml) for 24 h, MSCs were washed with PBS twice and then incubated with serum-free medium for the next 48 h.

HUVECs were seeded in 48-well plates coated with growth factor reduced Matrigel (BD Bioscience; 100 μl per well) at a concentration of 2 × 10^4^ cells per well and MSC-conditioned medium (300 μl per well) was added. Three wells were used for each sample of conditioned medium. Supplement-free and serum-free endothelial basal medium (EBM-2; Lonza) was utilized as a negative control. Plates were placed into a CO_2_-incubator at 37 °C and capillary-like structures were assayed at 6 h under a light microscope (Leica). The total length of the tubular structures was counted in five random fields of view per well using Photoshop CS5 software (Adobe, San Jose, CA, USA).

### Enzyme-linked immunosorbent assay (ELISA)

To ascertain whether omentin-1 pretreatment results in an increase in angiogenic humoral factor release from MSCs (vascular endothelial growth factor (VEGF), fibroblast growth factor-2 (FGF-2), hepatocyte growth factor (HGF), and insulin-like growth factor-1 (IGF-1)), MSCs were treated with omentin-1 (800 ng/ml) for 24 h and washed with PBS twice and then incubated with serum-free medium for the next 48 h. After that, the medium was collected. The levels of VEGF, FGF-2, HGF, IGF-1, interleukin-1 receptor antagonist (IL-1ra), tumor necrosis factor-stimulated gene-6 (TSG-6), interleukin (IL)-6, and IL-8 released from MSCs into the culture medium were directly measured by their respective ELISA kit according to the manufacturer’s instructions (R&D Systems Inc., Minneapolis, MN, USA; Sigma-Aldrich, MO, USA). Basal medium was used as a control. The absorbance was measured at 450 and 570 nm. To avoid the influence of cell numbers on cytokines levels, the data were normalized to cell counts. The assays were repeated three times.

### Reagent treatment

To investigate the role of the PI3K/Akt pathway in omentin-1-mediated MSC proliferation, and survival, proangiogenesis, and cytokine production, LY294002 (20 μm/L; Cell Signaling Technology) was added to the MSC medium for 1 h before omentin-1 treatment to block the PI3K/Akt pathway activation. All subsequent experiments were performed as described above.

### Statistical analysis

Data are expressed as mean ± standard deviation. Comparisons between two groups were measured using unpaired Student's two-tailed *t* test, and differences among groups were detected by one-way analysis of variance with Bonferroni post hoc test using the statistical software SPSS package v19.0 (SPSS, Inc., Chicago, IL, USA). *P* < 0.05 was considered significantly significant.

## Results

### Omentin-1 activated PI3K/Akt signaling pathway in MSCs

**Fig. 1:**
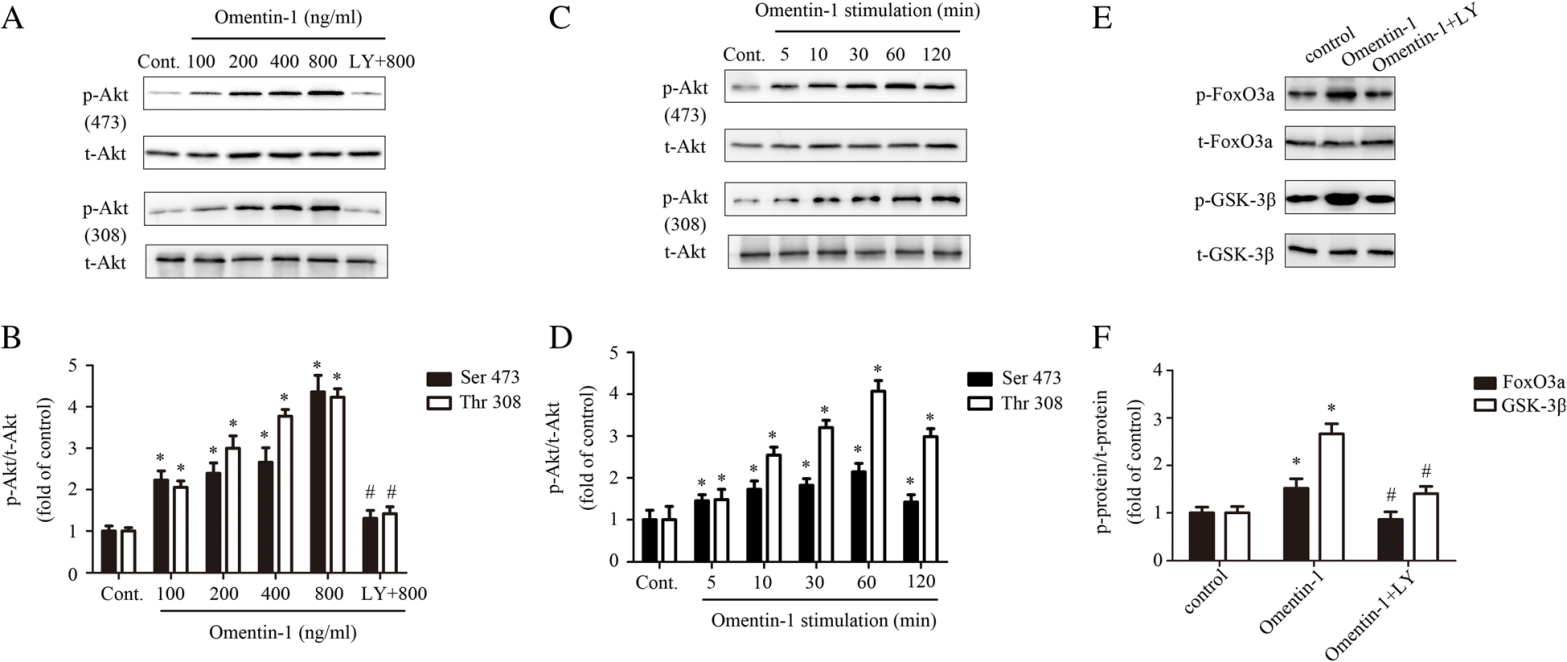
Omentin-1 activates the PI3K/Akt signaling pathway in a concentration- and time-dependent manner. The activation of the PI3K/Akt pathway was evaluated by Western blotting. **a**,**b** MSCs were incubated with different concentrations of omentin-1 (0–800 ng/ml) in serum-free medium for 1 h, or LY294002 (20 μm/L) was added to the MSC medium for 1 h before omentin-1 (800 ng/ml) treatment. **c**,**d** MSCs were treated with omentin-1 (800 ng/ml) in serum-free medium for the indicated periods (0–120 min). **e**,**f** Omentin-1 induced FoxO3a and GSK-3β phosphorylation via the PI3K/Akt pathway in MSCs. Data are presented as the mean ± SD of three separate experiments. *P < 0.05, versus the control group; ^#^ *P* < 0.05, versus the 800 ng/ml omentin-1 treated group. *Cont.* control, *LY* LY294002, *p-Akt* phosphorylated Akt (Ser473 and Thr308), *p-FoxO3a* phosphorylated FoxO3a, *p-GSK-3β* phosphorylated GSK-3β, *t-Akt* total Akt, *t-FoxO3a* total FoxO3a, *t-GSK-3β* total GSK-3β

Previous studies have shown that omentin-1 could activate the PI3K/Akt signaling pathway in various cells, such as macrophages [26], osteoblasts [27], vascular smooth muscle cells [28], endothelial cells [29], and cardiomyocytes [23]; this pathway plays an important role in MSCs. To further elucidate whether omentin-1 could activate the PI3K/Akt signaling pathway in MSCs, phosphorylation of Akt (Ser473 and Thr308) was detected by Western blot under increasing concentrations (0–800 ng/ml) or time-periods of stimulation (0–120 min) of omentin-1. As shown in Fig. 1a and b, omentin-1 treatment increased the levels of phosphorylated Akt (p-Akt) in a concentration-dependent manner. In the presence of the PI3K/Akt inhibitor, LY294002, p-Akt expression was strongly inhibited (Fig. 1a and b), indicating that omentin-1 was the upstream activator of the PI3K/Akt pathway in MSCs. Meanwhile, we observed that the expression of phosphorylated Akt proteins was enhanced by omentin-1 in a time-dependent manner, peaking at 60 min (Fig. 1c and d). FoxO3a and GSK-3β are downstream effectors of PI3K-Akt signaling [30, 31], and are well known to be implicated in regulation of MSC proliferation and survival [25, 32–34]. As shown in Fig. 1e and f, omentin-1 (800 ng/ml) exposure also caused a significant increase in the phosphorylation of FoxO3a and GSK-3β in MSCs. Furthermore, LY294002 abolished the phosphorylation of FoxO3a and GSK-3β subsequent to omentin-1 treatment (Fig. 1e and f).

### Omentin-1 promoted MSC proliferation via PI3K/Akt

**Fig. 2:**
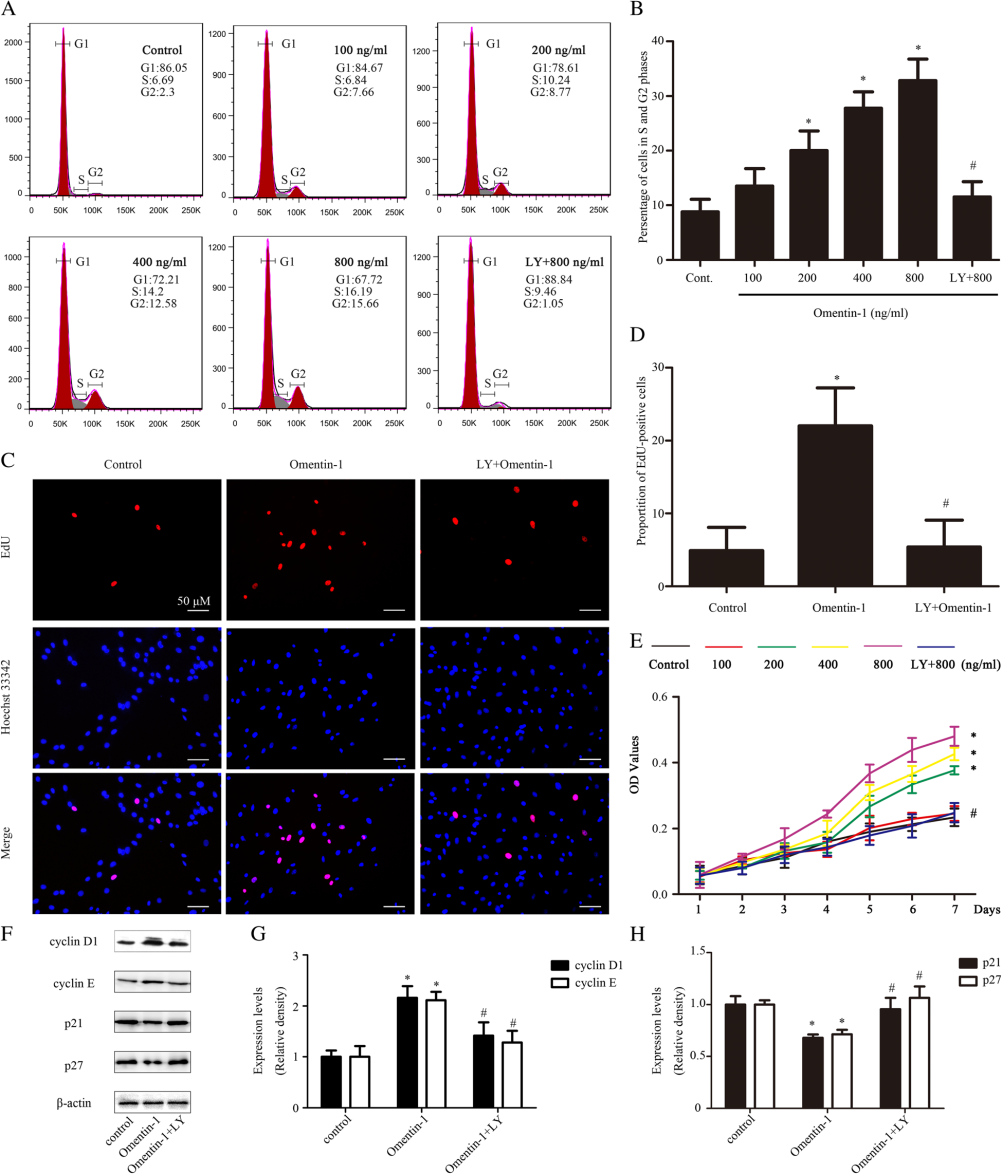
The PI3K/Akt signaling pathway was required for MSC proliferation induced by omentin-1. To determine the role of the PI3K/Akt signaling pathway in the proliferative actions of omentin-1, cells were pretreated with LY294002 (*LY*; 20 μm/L) before omentin-1 (800 ng/ml) treatment for 1 h. For the cell cycle assay and EdU assay, MSCs were treated with omentin-1 (0–800 ng/ml) with or without LY294002 for 5 days. **a**,**b** The cell cycle was evaluated using flow cytometry. **c**,**d** 5-Ethynyl-2’-deoxyuridine (EdU) assay showed that a blockade of PI3K/Akt could reduce omentin-1-mediated MSC proliferation. **e** Growth curve of MSCs after omentin-1 (0–800 ng/ml) intervention from days 1 to 7 with or without LY294002. **f**–**h** After treatment with omentin-1 (800 ng/ml) for 5 days, the protein expression levels of cyclin D1, cyclin E, p21, and p27 were markedly changed; however, they were abolished by pretreatment with LY294002. Data are presented as the mean ± SD of three separate experiments. *P < 0.05, versus the control group; ^#^ *P* < 0.05, versus the 800 ng/ml omentin-1 treated group. *Cont.* control, *OD* optical density

Experiments were carried out to investigate the effect of omentin-1 on MSC proliferation. The cell cycle, proliferation ability, and cell growth curve were tested by flow cytometry, EdU assay, and CCK-8, respectively. Varying doses of omentin-1 were added to the culture medium for 5 days following 24 h of serum starvation, and then flow cytometry showed the omentin-1 groups had a higher percentage of S and G2 phase cells compared with the control group in a concentration-dependent manner (Fig. 2a and b), while EdU-positive cells were significantly increased in the omentin-1 group (800 ng/ml) than in the control group (omentin-1 group, 22 ± 5 versus control group, 5 ± 3; *P* < 0.05; Fig. 2c and d). In line with flow cytometry and EdU assay data, the growth curves assessed by CCK-8 shown in Fig. 2e revealed that the growth ability of the MSCs improved gradually with increases in the omentin-1 concentration. However, there were no significant differences between the 100 ng/ml group and the control group (100 ng/ml group, 0.25 ± 0.02 versus control group, 0.23 ± 0.03; no significance; Fig. 5e). To further confirm whether the PI3K/Akt signaling pathway is essential to the proliferation effect of omentin-1 on MSCs, cells were preincubated with LY294002 (20 μm/L) for 1 h prior to omentin-1 treatment; we found that the PI3K/Akt inhibitor LY294002 dramatically suppressed this effect of omentin-1 on MSC proliferation (Fig. 2). These findings confirm that omentin-1 could promote MSC proliferation through the PI3K/Akt pathway in a concentration-dependent manner.

Cyclin D1 and cyclin E form the CDK4/6-cyclin D1 and CDK2-cyclin E complexes, which together promote the G1/S phase progression [35]. p21/p27 can bind to CDK2/4/6, playing a role in blocking the effect of cyclin-CDK complexes, and thus preventing G1/S phase transition [35]. Western blot results showed that the expression of cyclin D1 and cyclin E were significantly increased, while the expression of cyclin-dependent kinase inhibitors (CKIs) p21 and p27 were reduced markedly (Fig. 2f–h). However, pretreatment with LY294002 reversed this trend (Fig. 2f–h), suggesting that omentin-1 increased the proliferative capacity of MSCs through the PI3K/Akt pathway which promoted the cell cycle via a cyclin D1/E-p21/p27-dependent pathway.

### Omentin-1 inhibited H_2_O_2_-induced MSC apoptosis through PI3K/Akt

**Fig. 3:**
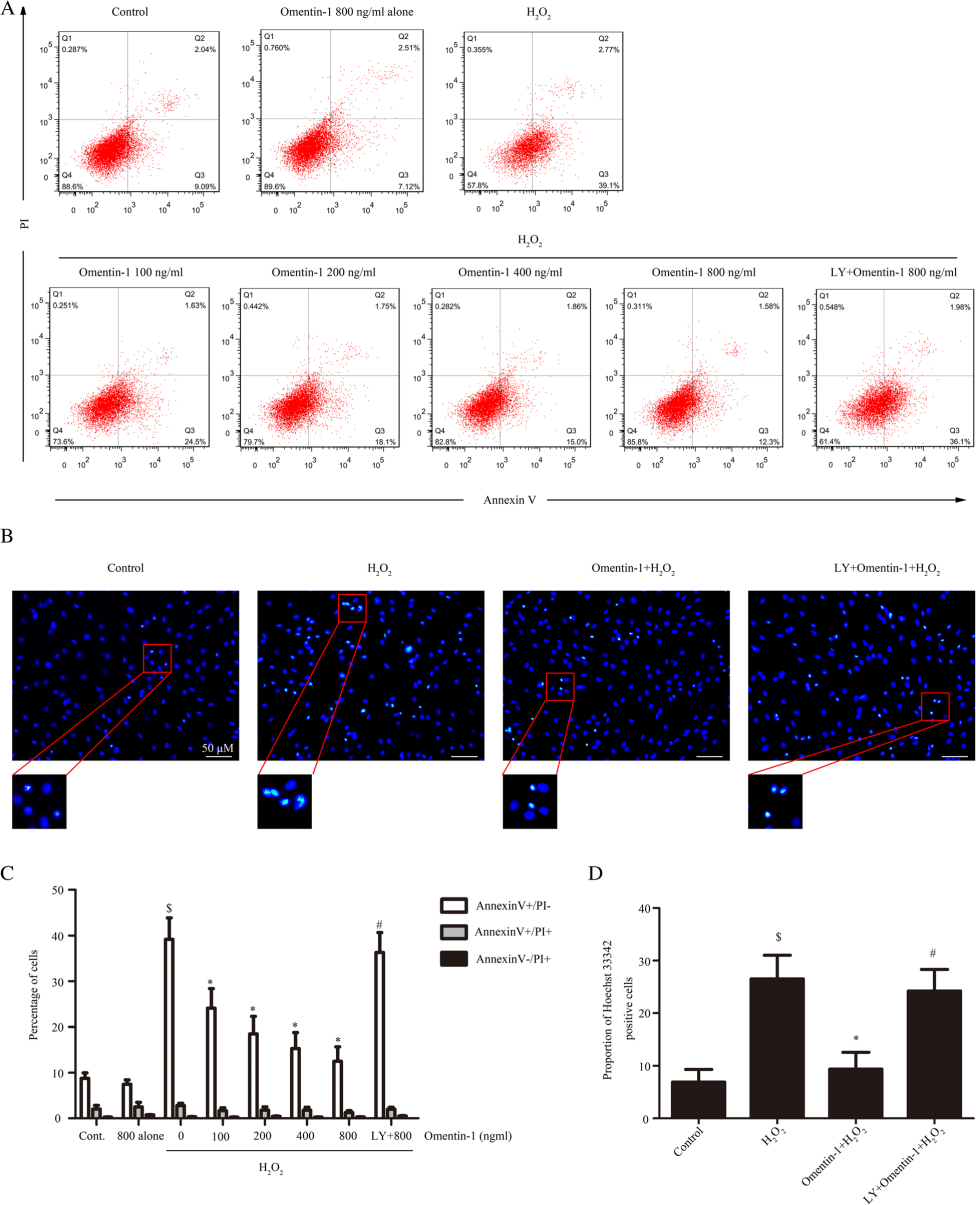
PI3K/Akt was involved in the antiapoptotic effects of omentin-1 on MSCs under oxidative stress. MSCs were pretreated with omentin-1 (0–800 ng/ml) for 1 h and continuously incubated with 400 μM H_2_O_2_ for 6 h. In order to identify the role of the PI3K/Akt signaling pathway on the antiapoptotic actions of omentin-1, LY294002 (*LY*; 20 μm/L) was added to the MSC medium for 1 h prior to omentin-1 (800 ng/ml) treatment. **a**,**c** Cell apoptosis was confirmed by Annexin V/propidium iodide (*PI*) and **b**,**d** Hoechst 33342 staining. Data are presented as the mean ± SD of three separate experiments. ^$^ *P* < 0.05, versus the control group; *P < 0.05, versus the H_2_O_2_ group; ^#^ *P* < 0.05, versus the 800 ng/ml omentin-1 treated group. *Cont.* control

Considering that apoptosis and necrosis caused by oxidative stress injury reduces the tissue repair capacity of MSCs [36], we examined the protective effects of omentin-1 on MSC death induced by H_2_O_2_. MSCs were pretreated with omentin-1 (0–800 ng/ml) for 1 h before being exposed to 400 μM H_2_O_2_ for 6 h, and the number of dead cells was evaluated by flow cytometry. H_2_O_2_ markedly increased the Annexin V^+^/PI^–^ cells (cells exposed to H_2_O_2_, 39.20 ± 4.7% versus normal cells, 8.76 ± 1.2%; *P* < 0.05; Fig. 3a and c), but not the Annexin V^+^/PI^+^ cells compared with the control group (cells exposed to H_2_O_2_, 2.27 ± 0.5% versus normal cells, 2.04 ± 0.8%; no significance; Fig. 3a and c), suggesting that H_2_O_2_ mainly induced the early apoptosis and had no effect on the late apoptosis or necrosis under our experimental conditions. When the MSCs were pretreated with omentin-1 for 1 h before H_2_O_2_, the percentage of Annextin V^+^/PI^–^ cells significantly decreased in a concentration-dependent manner. There was also no significant difference in cell apoptosis between the control cells and those pretreated with 800 ng/ml omentin-1 alone (Fig. 3a and c). To further confirm the antiapoptotic effects of omentin-1, morphological changes were observed using Hoechst 33342 staining. As seen in Fig. 3b and d, the data show that H_2_O_2_ increased cell apoptosis by approximately 20% (normal cells, 6.9 ± 2.4% versus cells exposed to H_2_O_2_, 26.5 ± 4.5%; *P* < 0.05) and the proportion of Hoechst 33342-positive cells was markedly decreased in the presence of omentin-1 (800 ng/ml group, 9.4 ± 3.2% versus cells exposed to H_2_O_2_, 26.5 ± 4.5%; *P* < 0.05). However, the protective effects of omentin-1 were attenuated when cells were preconditioned with LY294002 in both the flow cytometry test (LY294002 + 800 ng/ml group, 36.3 ± 4.4% versus 800 ng/ml group, 12.5 ± 3.2%; *P* < 0.05; Fig. 3a and c) and the Hoechst 33342 staining assay (LY294002 + 800 ng/ml group, 24.2 ± 4.1% versus 800 ng/ml group, 9.4 ± 3.2%; *P* < 0.05; Fig. 3b and d). These above results indicated that the PI3K/Akt signaling pathway played an important role in the protective effects of omentin-1 on H_2_O_2_-induced MSC apoptosis.

### Omentin-1 reduced ROS and protected the function of mitochondria in MSCs via PI3K/Akt

**Fig. 4:**
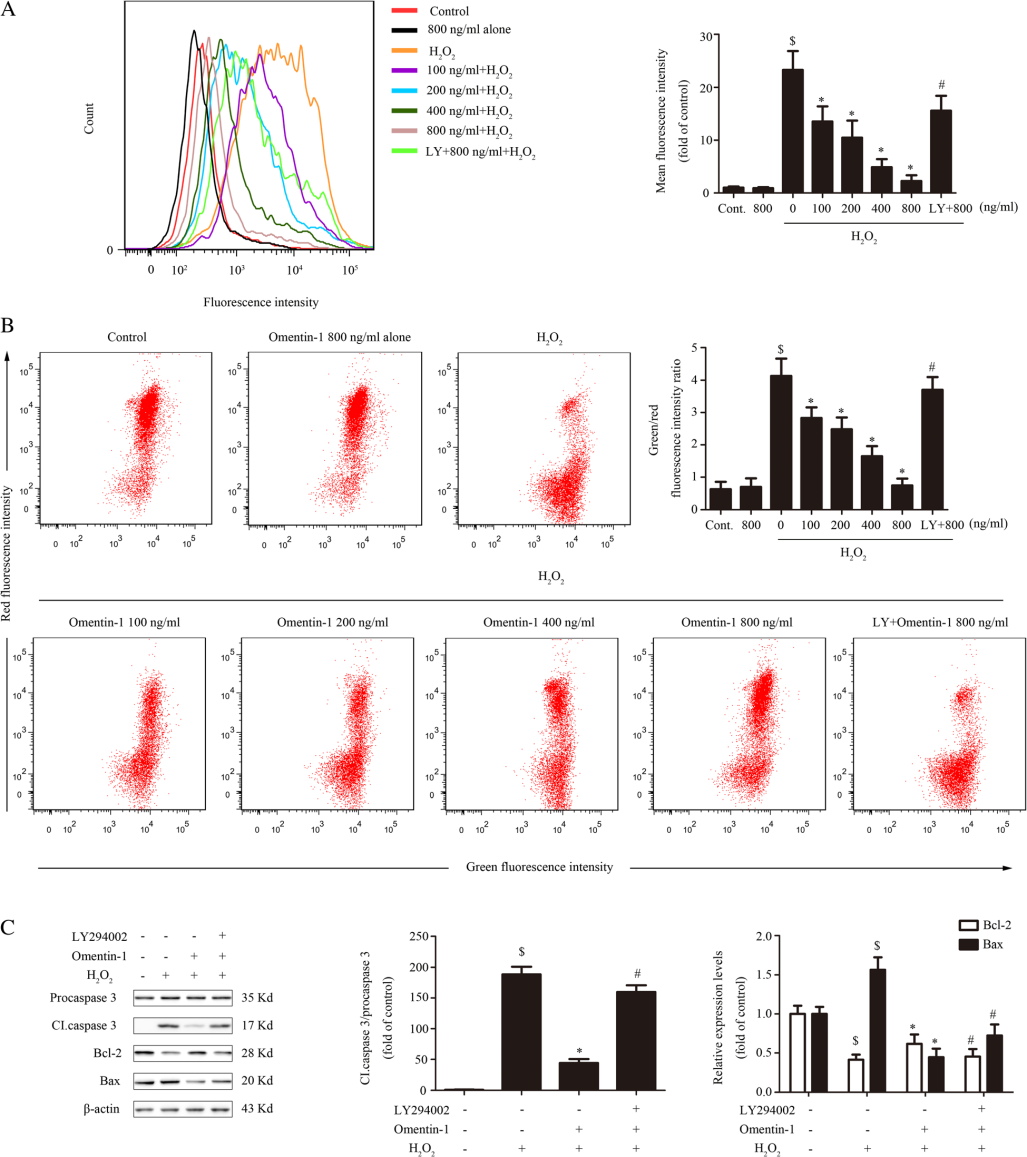
Effects of omentin-1 on ROS and the mitochondrial apoptosis pathway. MSCs were pretreated with omentin-1 (0–800 ng/ml) for 1 h and followed by exposure to H_2_O_2_ for 6 h. LY294002 (*LY*; 20 μm/L) was added into the MSC medium for 1 h prior to omentin-1 (800 ng/ml) treatment. **a** Intracellular ROS was assessed with DCFH-DA by flow cytometry. **b** The mitochondrial transmembrane potential of MSCs was analyzed by flow cytometry. **c** The expression of proteins associated with mitochondrial apoptosis pathways was evaluated by Western blotting. Data are presented as the mean ± SD of three separate experiments. ^$^ *P* < 0.05, versus the control group; *P < 0.05, versus the H_2_O_2_ group; ^#^ *P* < 0.05, versus the 800 ng/ml omentin-1 treated group. *Cl.caspase-3* cleaved caspase-3, *Cont.* control

Excessive exogenous ROS caused by H_2_O_2_ initiates cellular apoptosis that mainly depends on the mitochondrial death pathway whose progress involves the loss of mitochondrial membrane potential (ΔΨm), an imbalance in the Bax/Bcl-2 ratio, and activation of procaspase-3 [37]. Experiments were carried out to identify whether the antiapoptotic effect of omentin-1 on MSCs under H_2_O_2_ was dependent on reducing ROS and blocking the mitochondrial death pathway. First, the intracellular ROS level of MSCs was analyzed by DCFH oxidation assay. As shown in Fig. 4a, omentin-1 reduced the ROS level in MSCs after H_2_O_2_ treatment in a concentration-dependent manner, and was neutralized by LY294002. Furthermore, the mitochondrial transmembrane potential was measured by JC-1, a mitochondrial ΔΨm-sensitive dye. The results revealed that H_2_O_2_ gave rise to remarkable changes in mitochondrial ΔΨm compared with the control group (H_2_O_2_ group, 4.13 ± 0.53 versus control group, 0.63 ± 0.23; *P* < 0.05; Fig. 4b). However, pretreatment of MSCs with omentin-1 maintained an electrochemical gradient across the mitochondrial membranes in a concentration-dependent manner, which was also counteracted in the presence of LY294002 (Fig. 4b). Meanwhile, omentin-1 (800 ng/ml) alone had no significant effect on ROS levels and mitochondrial transmembrane potential (Fig. 4a and b). Western blot analysis was then used to measure the expression levels of cleaved caspase-3, procaspase-3, Bax, and Bcl-2, which are well known key mediators of apoptosis. Omentin-1 significantly suppressed the levels of cleaved caspase-3 and the ratio of Bax/Bcl-2 under H_2_O_2_ treatment (Fig. 4c). Consistently, the effects of omentin-1 on the proteins mentioned above were abolished by LY294002 (Fig. 4c).

Taken together, the data above suggest that pretreatment with omentin-1 could protect MSCs from exogenous oxidative stress and maintain the function of mitochondria in MSCs via PI3K/Akt.

### Omentin-1 elevated the ability of MSCs to stimulate tube formation by HUVECs and enhanced angiogenic growth factor secretion via PI3K/Akt

**Fig. 5:**
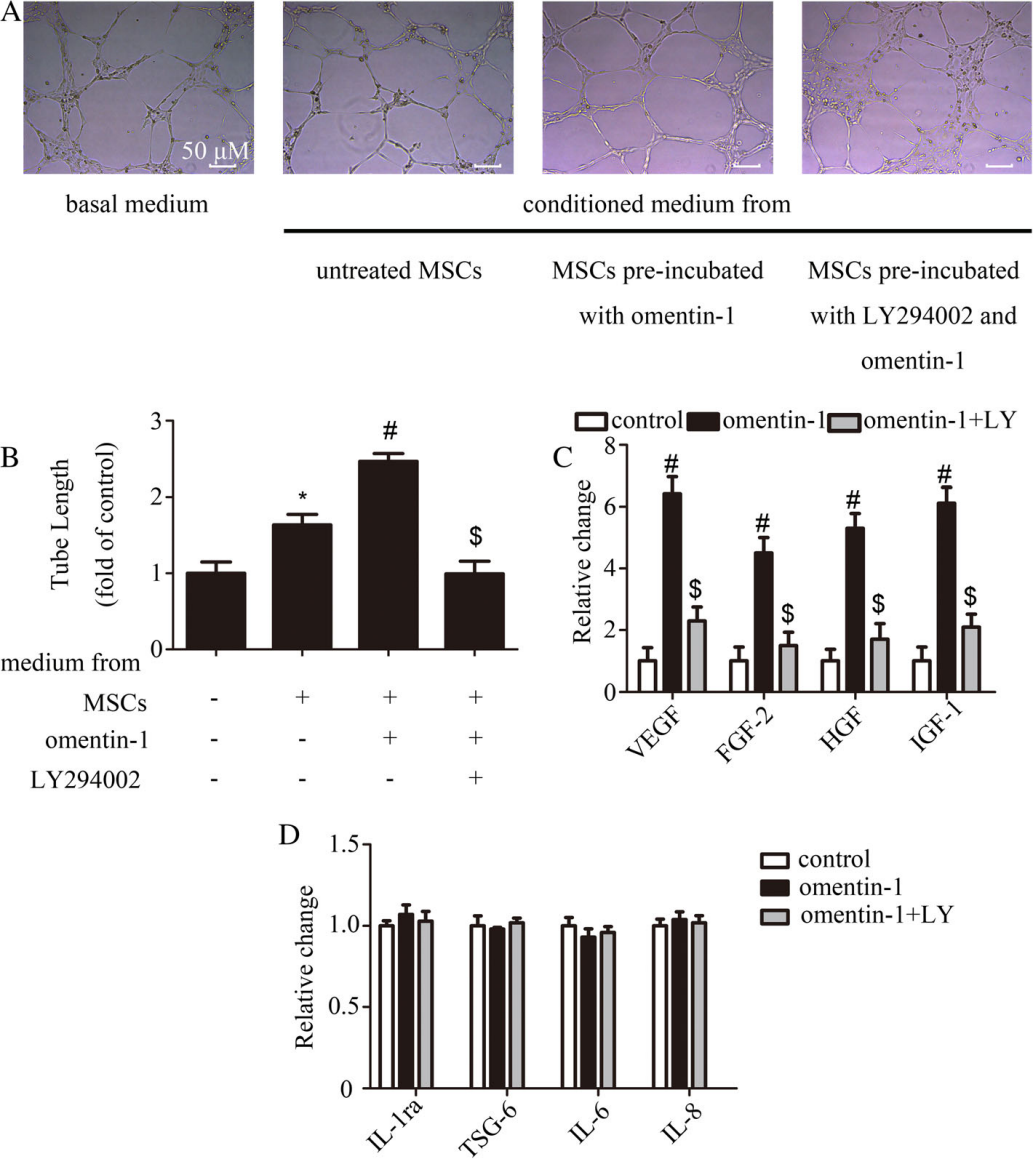
Effects of omentin-1 on the capillary-like tube formation capacity of the conditioned medium and angiogenic factor secretion. The media were conditioned by only mesenchymal stem cells (MSCs), omentin-1 (800 ng/ml) preincubated MSCs, or LY294002 (*LY*) and omentin-1 (800 ng/ml) preincubated MSCs. Then the conditioned media were collected. Basal medium was used as a negative control. **a**,**b** HUVECs were incubated in each conditioned medium for 6 h in Matrigel, and the total length of the tube network per field was quantified. **c** Concentrations of vascular endothelial growth factor (*VEGF*), fibroblast growth factor-2 (*FGF-2*), hepatocyte growth factor (*HGF*), and insulin-like growth factor-1 (*IGF-1*) in the conditioned medium were measured by ELISA and normalized to cell counts. **d** Concentrations of interleukin-1 receptor antagonist (*IL-1ra*), tumor necrosis factor-stimulated gene-6 (*TSG-6*), interleukin (*IL*)-6, and IL-8 in the conditioned medium were measured by ELISA and normalized to cell counts. Data are presented as the mean ± SD of three separate experiments. *P < 0.05, versus the basal medium; ^#^ *P* < 0.05, versus the conditioned medium from the untreated MSC group; ^$^ *P* < 0.05, versus the conditioned medium from the omentin-1-treated group

The tube formation assay was used to test whether conditioned medium from MSCs treated with omentin-1 could stimulate angiogenesis in vitro. MSCs were treated with omentin-1 (800 ng/ml) for 24 h with or without LY294002 and washed with PBS twice and then incubated with serum-free medium for the next 48 h. After that, the medium was collected. The results (Fig. 5a and b) indicate that conditioned media from untreated and omentin-1 (800 ng/ml) preincubated MSCs were able to augment endothelial cell capillary tube-forming capacity in vitro compared with basal medium and, in particular, the maximum proangiogenic effect was observed in the presence of conditioned medium from MSCs with preincubation with omentin-1, suggesting that the paracrine mechanism was involved in the proangiogenic effect of omentin-1 on MSCs. To further reveal this mechanism, the levels of VEGF, FGF-2, HGF, and IGF-1, as the key proangiogenic factors, were measured by ELISA. A statistically significant increase in all four factors mentioned above was detected in omentin-1 preincubated medium compared with the untreated medium (Fig. 5c). We then explored the role of the PI3K/Akt pathway in this mechanism. Pretreatment of MSCs with LY294002 attenuated the proangiogenic effect of omentin-1 as well as decreased the levels of VEGF, FGF-2, HGF, and IGF-1 in the conditioned medium (Fig. 5a–c). Meanwhile, various anti-inflammatory (IL-1ra and TSG-6) and proinflammatory (IL-6 and IL-8) cytokines from the medium had also been detected by ELISA. The results (Fig. 5d) did not show any significant change between groups. Moreover, ELISA was also used to assess whether MSCs could secrete rat omentin-1; however, we did not detect this in the MSC medium, and the results were further confirmed by wWestern blot and quantitative real-time polymerase chain reaction (qRT-PCR) in MSCs (data not shown), suggesting that MSCs might not be the source of omentin-1.

The data above suggest that omentin-1 could enhance the proangiogenic factor paracrine effect of MSCs via PI3K/Akt, thereby augmenting the endothelial cell capillary tube forming capacity in vitro.

## Discussion

In the present study, we investigated the effects of omentin-1 on MSCs and found that omentin-1 can promote MSC proliferation, inhibit H_2_O_2_-induced apoptosis, enhance angiogenic growth factor secretion, and elevate the ability of MSCs to stimulate tube formation by HUVECs. Moreover, these effects of omentin-1 were shown to be mediated, at least partially, through the PI3K/Akt signaling pathway. To our knowledge, this is the first study to report the beneficial effects of omentin-1 on MSCs and the underlying mechanisms.

Based on promising preclinical and clinical data, MSC therapy has been suggested as a potential therapeutic strategy for tissue repair [38]. Moreover, accumulating evidence suggests that MSCs repair the damaged tissue mainly by secreting cytokines and other paracrine factors to promote angiogenesis, activating endogenous reparative responses [39, 40] rather than directly by differentiation and numerical replacement of lost cell mass [40]. However, the success of MSC therapy still has its limitations. One of them is that the small number obtained from the donors requires extensive expansion for therapeutic utility [7, 8]. The other is that MSC therapy is compromised by the decreased survival of transplanted MSCs mainly due to oxidative stress and hypoxia in the harsh environment [9, 10], which in turn inhibits angiogenesis and tissue repair [41–43]. Therefore, to overcome these problems, researchers have attempted to enhance MSC proliferation, survival, and increase the secretion of cytokines for angiogenesis and other tissue repair function.

Omentin-1 is a new fat depot-specific secretory adipokine, the plasma levels of which are significantly decreased in patients with obesity, insulin resistance, diabetes, and cardiovascular diseases such as atherosclerosis, coronary artery disease, and ischemic heart disease [17, 20]. Although omentin-1 has been reported to exert proproliferation, prosurvival, and proangiogenic functions in various cells via an Akt-dependent mechanism [22, 23, 29], its specific role and underlying mechanism in MSCs remains unclear.

In the present study, the effects of different concentrations of omentin-1 (100, 200, 400, and 800 ng/ml) on MSCs were assessed. All of the concentrations of omentin-1, except 100 ng/ml, had a significant promoting effect on the growth of the MSCs in a time- and concentration-dependent manner. In addition, the number of cells in the S and G2 phases of the cell cycle significantly increased under omentin-1 exposure and the process was accompanied by upregulation of cyclin D1/E and downregulation of p21/p27. Of note, the concentration at 100 ng/ml was significantly lower than the physiological concentration in serum (200–400 ng/ml) [17, 44], suggesting that physiological and higher concentrations are required for MSC proliferation. Furthermore, we used H_2_O_2_ to induce oxidative stress to MSCs. The results showed that H_2_O_2_ induced higher intracellular ROS and more cellular apoptosis with a mitochondrial membrane potential decrease and caspase-3 activation. Moreover, excessive ROS could attack the mitochondrial membrane, leading to loss of the potential of the mitochondrial membrane and the consequent apoptosis of MSCs [45]; however, omentin-1 pretreatment could reduce the excessive ROS, restore mitochondrial membrane potential, and inhibit the apoptosis of MSCs in a concentration-dependent manner. In addition, the balance of Bax and Bcl-2 is responsible for the integrity of the mitochondrial membrane and ΔΨm stabilization [46]. Higher Bax and lower Bcl-2 levels resulted in a loss of ΔΨm and mitochondrial swelling or disruption [46]. In contrast, our experiment demonstrated that omentin-1 pretreatment upregulated Bcl-2 but downregulated Bax, which contributed to the preservation of mitochondrial function. Caspases are downstream of the Bcl-2 family in the apoptotic cascade and caspase-3 is one of the key effectors of apoptosis [47]. We further detected the activation of caspase-3 and found that omentin-1 pretreatment markedly reduced the levels of cleaved caspase-3 compared with the cells treated with H_2_O_2_ alone. Therefore, these findings suggest that omentin-1 may exert its protective property in oxidative stress-induced MSC apoptosis by reducing the excessive ROS and inhibiting mitochondria-dependent caspase cascades. Furthermore, our data showed that omentin-1 could increase MSC phosphorylated Akt levels in a time- and concentration-dependent manner. However, LY294002, the specific inhibitor of PI3K, not only blocked the activation of Akt induced by omentin-1, but also significantly attenuated the promoting effects of omentin-1 on the growth and survival of MSCs, suggesting that omentin-1 treatment activated Akt and led to MSC proliferation and survival.

Forkhead box O3 (FoxO3a), a transcription factor, is the downstream target of Akt [48]. Moreover, FoxO3a can promote antiproliferation or proapoptosis signaling through either increasing the protein levels of cyclin-dependent kinase inhibitors [49], or regulating the expression of Bcl-2 family proteins [50]. Activated Akt-mediated phosphorylation and inactivation of FoxO3a improves cell proliferation and survival. Likewise, glycogen synthase kinase-3β (GSK-3β) also exists downstream of Akt and acts as a key regulator of multiple processes that are critical for the proliferation and apoptosis in various cells, including MSCs [51, 52]. In this present study, we found that FoxO3a and GSK-3β were phosphorylated by omentin-1, which might be a direct result of Akt activation, and this was reversed in the presence of LY294002. This suggested a potential role of FoxO3a and GSK-3β in omentin-1-induced MSC proliferation and survival.

In addition, omentin-1 has also been observed to exhibit antiproliferative and proapoptotic effects via the PI3K/Akt pathway in other types of cells, such as human osteoblasts [27], mouse neural stem cells [53], cardiomyocytes [23], and endothelial cells [22]. In contrast, Zhang and colleagues [54] provided evidence that omentin-1, as an anticancer factor, inhibited human hepatocellular carcinoma cell proliferation and survival via the JNK signaling pathway. It was also found that omentin-1 attenuated neointimal formation after arterial injury and suppressed vascular smooth muscle cell proliferation through AMPK-ERK-dependent mechanisms [55]. One possible explanation for this discrepancy could be that the activity of these signaling pathways is differentially controlled in specific cells in response to omentin-1 treatment, and the specificity of the regulation is dependent on cell type and/or its exposure environment.

During the past decade, it has been demonstrated that MSCs can promote angiogenesis by the secretion of proangiogenic factors (e.g., VEGF, IGF-1, HGF, and FGF-2) that contribute to tissue repair and enhance the reparative process [41, 56, 57]. Thus, various strategies have been adopted to upregulate the secretion of angiogenic factors in MSCs, such as gene transfer [41] and culture under hypoxic conditions [58]. Although it has also been reported that omentin-1 could directly promote endothelial cell function and revascularization in the hind limb ischemia mouse model [22], the indirect effect of omentin-1 on angiogenesis through MSCs remains unclear. In this study, we observed that conditioned medium from omentin-1-pretreated MSCs significantly increased the tube length relative to the conditioned medium from untreated MSCs and basal medium. Furthermore, we confirmed that proangiogenic factors (VEGF, FGF-2, HGF, and IGF-1) from omentin-1-pretreated MSC conditioned medium had a higher level than that from untreated MSCs; thus we concluded that omentin-1 could augment endothelial cell capillary tube forming capacity in vitro at least partially due to the upregulation of the secretion of angiogenic factors by MSCs. Additionally, a blockade of the Akt pathway caused a reduction in the tube-forming capacity and angiogenic cytokines of conditioned medium from MSCs pretreated with omentin-1. Together, these data revealed that the proangiogenic effects of omentin-1 on MSCs are mediated at least partially through the PI3K/Akt signaling pathway. However, these results do not rule out the proangiogenic effects of other factors that are secreted by MSCs, such as Ang-1, Ang-2, IL-6, and PLGF. It will be interesting to acquire more information on the angiogenic factors found in the MSC secretome induced by omentin-1. In addition to proangiogenic factors, we also detected several main anti-inflammatory (IL-1ra and TSG-6) and proinflammatory (IL-6 and IL-8) cytokines from the MSC medium; however, there was no significant change between groups, suggesting that omentin-1 might at least have no effects on the secretion of the inflammation-associated cytokines mentioned above by MSCs.

Of note, the signaling pathways mediating the protective effects of omentin-1 are complex and never exclusive. Several studies have argued that AMPK could be activated by omentin-1 and involved in the regulation of proliferation and apoptosis [22, 23, 59]; whether this pathway also contributes to the beneficial influence of omentin-1 and its possible crosstalk with Akt require further research. Moreover, contributions of other adipokines, especially those that exert positive effects on health such as C1q/TNF-related protein 9 (CTRP9), must be elucidated in future studies.

## Conclusion

Overall, the results of the present study provide preliminary evidence indicating that omentin-1 has beneficial effects on MSCs by promoting proliferation, inhibiting apoptosis, increasing secretion of angiogenic cytokines, and enhancing the ability for stimulating tube formation by HUVECs. In addition, the beneficial effects of omentin-1 are possibly a result of the activation of the PI3K/Akt signaling pathway. These data collectively demonstrate that omentin-1 may be considered a candidate for optimizing MSC-based cell therapy.

## Abbreviations

AMPK: AMP-activated protein kinase
CCK-8: Cell counting kit-8
CTRP9: C1q/TNF-related protein 9
DCFH-DA: 2',7'-Dichlorodihydrofluorescein diacetate
DMEM/F12: Dulbecco's modified Eagle’s medium/nutrient mixture F-12
EdU: 5-Ethynyl-2’-deoxyuridine
ELISA: Enzyme-linked immunosorbent assay
FGF-2: Fibroblast growth factor-2
FoxO3a: Forkhead box O3
GSK-3β: Glycogen synthase kinase-3β
HGF: Hepatocyte growth factor
HUVEC: Human umbilical vein endothelial cell
IGF-1: Insulin-like growth factor-1
IL: Interleukin
IL-1ra: Interleukin-1 receptor antagonist
MSC: Mesenchymal stem cell
p-Akt: Phosphorylated Akt
PBS: Phosphate-buffered solution
PI: Propidium iodide
PVDF: Polyvinylidene difluoride
ROS: Reactive oxygen species
SD: Sprague-Dawley
SDS-PAGE: Sodium dodecyl sulfate-polyacrylamide gel electrophoresis
TBS: Tris-buffered saline
TBS-T: TBS with Tween-20
TSG-6: Tumor necrosis factor-stimulated gene-6
VEGF: Vascular endothelial growth factor
ΔΨm or MMP: Mitochondrial membrane potential
